# Wearable Continuous Vital Sign Monitoring Study (WARD-AMS) to Detect Clinical Deterioration in Postoperative General Surgery Patients: Protocol for a Randomized Controlled Trial

**DOI:** 10.2196/81558

**Published:** 2025-12-19

**Authors:** Jasjit Syan, Meera Joshi, John Beard, Jonah Attebery, Fu Siong Ng, Sadia Khan

**Affiliations:** 1 National Heart and Lung Institute, Imperial College London, ICTEM, Hammersmith Campus, London, W12 0NN, United Kingdom, 44 2075942739; 2 Department of Cardiology, Chelsea and Westminster NHS Foundation Trust, London, United Kingdom; 3 Department of Surgery and Cancer, Imperial College London, London, United Kingdom; 4 Patient Care Solutions, GE HealthCare, Chicago, IL, United States

**Keywords:** ambulatory monitoring, continuous vital sign monitoring, patient deterioration, postoperative care, wearable sensors, health care technology, digital health

## Abstract

**Background:**

Postoperative complications remain a major challenge in health care systems, with more than 70% of postoperative in-hospital mortality occurring in general wards. Current practice relies on intermittent spot-check monitoring of vital signs guided by track-and-trigger thresholds such as early warning scores. Evidence shows that, up to 4 hours before an adverse event, abnormal vital signs can be detected in up to 60% of patients. Continuous vital sign monitoring in general wards offers the potential to detect clinical deterioration earlier. However, prospective evaluations of continuous monitoring in general wards remain limited.

**Objective:**

This study aims to prospectively evaluate the impact of continuous vital sign monitoring in adult postoperative patients in general wards. The primary objective is to determine whether continuous vital sign monitoring reduces the time from device-defined physiological deterioration to the first objective escalation event (acknowledgment or clinical action) by the health care team. Secondary objectives are to evaluate clinical outcomes, operational impact, and both staff and patient experiences.

**Methods:**

We conducted a single-center, open-label randomized controlled trial of 200 general surgical patients, receiving continuous vital signs monitoring alongside standard care. Participants were randomized 1:1 to alerting or nonalerting groups, either real-time data and alerts visible or blinded to clinicians, respectively. The primary outcome is the time from the first device-defined physiological deterioration episode to the first objective escalation event (acknowledgment or clinical action). Exploratory outcomes evaluate time to treatment initiation, rapid response team activation, escalation of care, length of stay, mortality, alarm performance, and patient and staff experience.

**Results:**

The Ward Alerting and Ambulatory Monitoring Study (WARD-AMS) trial received ethical approval in April 2024; recruitment commenced in October 2024 and was completed by December 2025 with 200 patients enrolled. Data analysis is ongoing, with results to be submitted for publication by mid-2026.

**Conclusions:**

The WARD-AMS study is among the first prospective, randomized evaluations of continuous vital sign monitoring in real-world ward settings looking at clinical outcomes and user experience. By integrating clinical outcomes, alarm analytics, and stakeholder experiences, it aims to provide a comprehensive understanding of the benefits, limitations, and implementation challenges of continuous monitoring. Findings from this study will inform strategies to optimize response to early clinical deterioration, reducing alarm fatigue and embedding new technologies into existing routine ward practice.

## Introduction

Despite significant advancements in surgical techniques and perioperative care, postoperative complications continue to present substantial clinical challenges, negatively impacting both patient health and health care systems. Findings from the European Surgical Outcomes Study revealed that 73% of patients who died in hospital were never admitted to an intensive care unit (ICU) [1], whereas another study added that general wards experienced the highest rate of adverse events, followed by operating theaters and ICUs at 48.8%, 26.1%, and 13%, respectively [2]. Additionally, it has been shown that, up to 4 hours before cardiorespiratory arrest, 59.4% of patients have at least one abnormal vital sign and there is a stepwise increase in mortality for patients with an increasing number of abnormal vital signs [3]. Current standard-of-care intermittent monitoring protocols suggest measuring vital signs every 4 to 8 hours in a general ward. There is a sharp drop in surveillance intensity from continuous monitoring intraoperatively and in ICUs to intermittent manual checks in the ward, where clinical deterioration may go undetected [4]. Despite repeated evidence highlighting the critical role of respiratory rate as a marker of clinical deterioration, inaccuracies in manual measurements persist, with no improvements in technology to support bedside nursing practice [5-7].

Continuous vital sign monitoring systems, successfully used in ICUs, offer a promising strategy to extend enhanced surveillance to general wards, addressing some of these issues. Recent innovations have led to the development of wireless, wearable continuous vital sign monitoring devices, mitigating previous barriers such as size and restrictive tethering [8]. It is known that early mobilization within the first 24 hours after surgery promotes improved lung function, reduces thromboembolic risk, and shortens hospital stay [9,10]. Wearable vital sign monitors facilitate this by allowing for continued surveillance without tethering patients to bedside monitors [11,12]. Nevertheless, early retrospective and limited prospective before-and-after analyses have reported modest clinical benefit of these technologies when applied in general wards [13]. These observations demonstrate significant barriers related to device implementation and integration into existing health care systems. A recent systematic review and meta-analysis of randomized controlled trials looking at the outcomes of patients with continuous monitoring of vital signs in general wards demonstrated nonsignificant trends for improvements in in-hospital mortality, major events or complications, or higher-level care admission, with pooled odds ratios of 0.95, 0.71, and 0.82, respectively, and a mean reduction in length of stay of 2.12 days [13]. The randomized controlled trials evaluated were constrained by the heterogeneity of monitoring technologies (devices measuring diverse combinations of vital signs and varying alert parameters) and the inconsistent definitions of major complications. In addition, by not comparing with a standardized escalation pathway (who is exposed to the alarms and data, at which thresholds, and how does staff intervene), the review overlooked the critical influence of response workflows, limiting insight into whether outcomes reflect technology performance or differences in clinical practice. Understanding of how nursing and health care staff navigate the challenges of increased data volume, alarm fatigue, and changes in workflow must be realized to successfully use these technologies to improve morbidity and mortality beyond the ICU [14,15]. Additionally, patient knowledge, attitude, and perceptions regarding digital devices have been incompletely explored, lacking validation and weakened by selection bias [16-18].

This study aims to evaluate whether continuous vital sign monitoring reduces the time from device-defined physiological deterioration (T_0_) to the first objective escalation event (T_action), defined as acknowledgment or clinical action by the health care team, compared with the standard of care (intermittent monitoring) in postoperative adult patients in general surgical wards. Secondary aims are to evaluate the effect of continuous vital sign monitoring on escalation of care, timely intervention, and corresponding clinical outcomes. By capturing and analyzing the perspectives of nurses and patients, key stakeholders in device implementation, we seek to identify practical barriers and facilitators, thereby proposing actionable solutions to facilitate effective integration of continuous vital sign monitors into routine ward practice and enhance patient outcomes without imposing a burden upon health care professionals.

## Methods

### Study Design

A prospective, interventional randomized controlled trial will be conducted using a mixed methods approach. It is an open-label, 2-arm study that aims to recruit 200 patients over a 1-year period. Patients will be randomized using an online tool (Greenlight Guru Clinical) on a 1:1 basis into an alerting and nonalerting group ( Figure S1 in Multimedia Appendix 1). This study will be conducted on 2 postsurgical wards at the West Middlesex University Hospital, London, with a capacity of 64 beds, providing orthopedic, general surgical, urological, gastroenterological, and gynecological care.

Patients are screened after the morning ward round, and those who are expected to remain in hospital for at least 1 night are approached by the research team for consent and assessment against the inclusion and exclusion criteria. Most of these patients are postoperative, although a small number are preoperative. All patients will be fitted with a continuous vital sign monitor (Portrait Mobile; GE HealthCare) from recruitment until either discharge from hospital or transfer to another unit. This CE-marked device (European Union certificate CR-03-1004-804-22) consists of 2 wearable sensors: a wrist-mounted saturation probe measuring both the oxygen saturation and pulse rate and a chest sensor measuring the respiratory rate. Both groups will have vital signs monitored as per the standard of care, intermittently acquired every 4 to 6 hours by health care staff using the National Early Warning Score 2 for identification and escalation of a deteriorating patient [19].

Only the alerting group will have their continuous vital signs transmitted to a visible central viewer dashboard located at the nursing station and have audible alarms enabled on both the bedside hub monitor and the central viewer. All ward health care staff have access to real-time patient data and alarms; however, the nurse in charge is responsible for recognizing alarms, logging them, and informing the bedside nurse to respond appropriately. The intervention in this trial is the activation of real-time alerting through the continuous vital sign monitor, and alarms are managed entirely by the usual ward clinical team. They follow standard hospital escalation protocols, escalating deteriorating patients to the ward physician during daytime hours, the on-call physician out of hours, or the critical care outreach team. The critical care outreach team (also known as the rapid response team [RRT]) is an existing hospital team of specialized nurses who respond to acutely unwell patients requiring urgent assessment or intervention outside of the ICU. In the nonalerting group, health care staff are blinded to the continuous monitoring data.

To address alarm fatigue, several considerations have been taken. The alerting thresholds and announcement delays were derived from the Continuous Vital Sign Monitoring in Surgical Wards (COSMOS) pilot study, which conducted its initial phase by analyzing alarm data (pulse rate of <45 beats per minute [bpm] or >130 bpm sustained for ≥30 seconds, respiratory rate of <4 breaths per minute sustained for ≥30 seconds or >30 breaths per minute sustained for ≥120 seconds, apnea sustained for ≥30 seconds, and peripheral oxygen saturation of <85% sustained for ≥90 seconds or <80% without delay) [20]. These clinically validated alarm thresholds and built-in delay minimize false alerts caused by artifacts or transiently deranged vital signs. The central viewer is stationed at the nursing bay, beside the desk of the nurse in charge, who triages alarms. In addition, all ward staff received structured device training from GE HealthCare representatives focusing on correct interpretation, prioritization, and escalation of alarms.

Blinding of clinicians was not feasible as the intervention requires visible and audible alarms in real time to be recognized and acted upon by ward teams. The purpose of this study is to evaluate continuous vital sign monitoring in existing ward workflows, where staff awareness of alarms is intrinsic to the intervention itself. Several design features were incorporated to minimize performance and detection bias: all patients will receive standard-of-care monitoring, staff training was delivered similarly across both trial arms, and the primary end points were derived objectively from either device or electronic patient record (EPR) time stamps rather than relying on subjective documentation. Although some degree of behavior modification is unavoidable, these measures attempt to minimize bias.  

### Sample Size

In a pilot study, approximately 15% of patients wearing the continuous vital sign monitor generated at least one physiological alarm, with an average response time of 154 minutes [21]. In this trial, we defined a clinically meaningful target as a reduction in response time from 154 minutes to 60 minutes, corresponding to an estimated hazard ratio of 2.6 for time to response between the alerting and nonalerting groups. Therefore, recruiting a minimum of 200 patients, 100 in each arm, yields at least 30 participants with analyzable alert events. This is sufficient in providing reasonable precision for estimating the primary effect size and to support analyses of secondary outcomes.

In a previous feasibility study, 969 respiratory rate alerts were generated from 276 patients, supporting the assumption that a sample size of 200 patients generates sufficient signal volume for detailed alarm analyses [21].

### Participants

Patients are identified and assessed for eligibility based on several criteria. Inclusion criteria are patients aged >18 years who provide informed consent to be part of the study. Exclusion criteria are patients aged <18 years and those who are unable to provide informed consent, have an allergy to the electrocardiogram or sensor electrodes or skin conditions at the sensor site, have an implantable electronic cardiac device (which responds to minute ventilation and can interfere with respiratory rate measurement technology), or are already taking part in another study.

Nursing staff, including both registered nurses and health care assistants, are invited to participate in the study. After recruiting 100 patients, all staff on the delegation log will be invited to complete a structured questionnaire, and a subset who volunteer will be selected for a follow-up interview at the end of the study period.

### Ethical Considerations

All participants will be required to provide informed consent. Ethics approval was granted by the Health Research Authority and Health and Care Research Wales (approval reference 24/YH/0073; Integrated Research Application System project ID 335808) on April 18, 2024. This study is conducted in accordance with the principles of good clinical practice, the UK policy framework for health and social care research, and the Declaration of Helsinki. All patient data will be anonymized, and patients will be assigned a unique study ID. No identifiable patient data will be shared with a third party.

### Trial Registration

The trial is registered with the International Standard Registered Clinical/Social Study Number (ISRCTN15518094).

### Outcomes

Despite the growing interest in continuous vital sign monitoring, most prospective trials are limited by assessing only feasibility and acceptability and are not always powered for patient-centered outcomes [20,22-29]. There is still a clear lack of understanding of how continuous physiological data translate into alerts, how these alerts influence clinician assessment and intervention, and whether these actions ultimately improve patient outcomes. The WARD-AMS study design assesses the full monitoring-alert-action-outcome pathway.

### Primary End Point

The primary end point is the time (in minutes) from the first device-defined deterioration episode (T_0_) to the first objective escalation event (T_action) among patients who experience at least one device-defined deterioration episode during the monitoring period.

#### Definition of T_0_ (Time Zero)

T_0_ is defined as the time stamp of the first device-defined deterioration episode for each patient according to the prespecified WARD-AMS deterioration criteria: pulse rate of <45 bpm or >130 bpm sustained for ≥30 seconds, respiratory rate of <4 breaths per minute sustained for ≥30 seconds or >30 breaths per minute sustained for ≥120 seconds and apnea sustained for ≥30 seconds, and peripheral oxygen saturation of <85% sustained for ≥90 seconds or <80% without delay.

#### Definition of T_Action (Escalation Event)

T_action is defined as the earliest time stamp after T_0_ at which any of the following prespecified escalation events occurs:

- Activation of an RRT call: time of official call log in the switchboard or outreach database
- Unplanned transfer to higher level of care: time of actual bed movement from the ward to the ICU, high-dependency unit (HDU), or critical care unit as recorded on the EPR
- New high-acuity treatment orders (time stamp of order entry on the EPR): new intravenous (IV) fluid; new start of vasopressor or inotrope; new start of high-flow oxygen, continuous positive airway pressure, or noninvasive ventilation; and new start of broad-spectrum IV antibiotics
- Time stamp of alarm acknowledgment on the nursing log

For each patient with at least one eligible deterioration episode, only the first deterioration episode (first T_0_) will be included in the primary analysis. T_action is the earliest qualifying escalation event occurring after that T_0_. If at least one escalation event occurs after T_0_, the primary outcome is calculated as follows and expressed in minutes:


$$\text{Primary outcome}=T_{\text{action}}-T_{0}$$

If no escalation event occurs before censoring, the observation is right censored at the prespecified censor time (eg, end of monitoring period, discharge, death, or withdrawal from the study).

Acknowledgment of clinical deterioration can occur through several legitimate pathways in routine ward practice; therefore, relying on a single indicator may risk meaningful clinical response detection. To address this, a composite definition of T_action is chosen to ensure that the earliest objective, time-stamped event is captured. The same criteria are applied across both trial arms.

### Exploratory End Point

Exploratory end points will further evaluate a broader range of clinical, operational, alarm-related, and user experience outcomes. Time-to-event clinical end points include time from T_0_ to initiation of key treatments, such as antibiotics, IV fluids, vasopressors, oxygen, or advanced respiratory support. Additional timing end points include the interval from T_0_ to RRT activation and from T_0_ to unplanned transfer to an HDU or ICU. Resource use outcomes will be explored, including length of hospital stay, rates of admission to higher-level care, frequency of RRT activation, and in-hospital mortality.

Analysis of alarms will include identifying the frequency, type, and clinical relevance of alerts triggered by the continuous vital sign monitor, as well as the proportion of alarms that are associated with escalation events. Patient and health care staff experience will be evaluated via structured questionnaires and semistructured interviews.

### Health Care Staff Questionnaire and Interview

An adapted System Usability Scale questionnaire will be used to evaluate health care staff experiences with the Portrait Mobile system (Figure S2 in Multimedia Appendix 1). This adaptation is based on a validated framework designed to quantify user experience and acceptance [30]. Consent will be obtained before staff participation, and the questionnaire will be completed online on an electronic form via Greenlight Guru [31]. Responses will be captured using a 5-point Likert scale ranging from “strongly agree” to “strongly disagree,” allowing for precise measurement of attitudes toward regular use, complexity, integration of functions, and overall usability. Open-ended questions at the end will permit staff to share any specific experiences or suggestions for improvement, enhancing qualitative understanding of the quantitative findings.

Semistructured interviews will be conducted using a predefined guide of questions formulated following a literature review (Figure S3 in Multimedia Appendix 1). The guiding questions follow themes regarding system functionality, patient safety implications, effectiveness, adequacy in training, practical barriers and facilitators, technological challenges, and suggestions for system enhancements. These interviews will be audio recorded, anonymized, and transcribed verbatim, followed by analysis using NVivo (version 12; Lumivero) [32].

### Patient Questionnaire and Interview

At least 24 hours after being recruited to the trial, patients will complete a questionnaire to assess their perceptions and experiences of the Portrait Mobile system (Figure S4 in Multimedia Appendix 1). This aims to collect information on comfort, usability, perceived impact on health care outcomes, and overall satisfaction with the monitoring device.

Additionally, a follow-up telephone interview will be conducted approximately 2 weeks after discharge to evaluate longer-term perceptions and outcomes (Figure S5 in Multimedia Appendix 1). These interviews are structured to capture detailed information on patient experiences and suggested improvements.

### Statistical Analysis

#### Primary End Point Analysis

Among the 2 groups (blinded and unblinded), the primary comparison will be the time from device-defined deterioration to objective escalation. The primary analysis will be performed in the population of patients who experience at least one device-defined deterioration episode, restricted to only 1 episode per patient (the first). Time-to-event methods will be used, with Kaplan-Meier curves constructed for each arm to visualize the distribution of time to escalation. The primary hypothesis test will use a log-rank test comparing the survival curves between arms, and a Cox proportional hazard model will be fitted with the interventional arm as the main exposure to estimate the hazard ratio for escalation (intervention vs control) with corresponding 95% CIs. The Cox model will be adjusted for key baseline covariates (age, sex, and comorbidity burden [Charlson Comorbidity Index]).

Patients with a device-identified deterioration without an escalation event during follow-up until their censor time will be treated as right censored in all time-to-event analyses. Proportional hazard assumptions will be evaluated using standard diagnostics such as Schoenfeld residuals. If these assumptions are materially violated, alternative or supplementary summaries such as restricted mean time to escalation up to a predefined time horizon may be reported in sensitivity analyses.

#### Exploratory Analysis

Analysis of all clinical end points involving time-to-event data will be conducted using survival analysis methods, specifically Kaplan-Meier curves and log-rank tests to assess differences between the alerting and nonalerting arms.

Length of hospital stay will undergo additional analysis using nonparametric tests to evaluate differences in median length of stay between the 2 study arms. Secondary outcomes presented as categorical variables, such as frequency of HDU or ICU admission and RRT activation, will be compared using chi-square tests. These will be conducted using RStudio (Posit PBC) [33].

Quantitative data from both the health care staff and patient questionnaires will be analyzed to illustrate frequency distributions and central tendencies regarding system usability, satisfaction, and other outcome measures. Likert-scale responses will be numerically scored and statistically analyzed using RStudio [33].

Interview transcripts will be analyzed by generating a set of codes that will be predefined from existing literature and serve as initial labels to index relevant data segments. This will be refined through iterative coding and thematic consolidation to ensure accurate and meaningful categorization by merging similar codes and splitting overly broad codes. To enhance validity, this method will be performed by 2 independent researchers until thematic consensus and data saturation are reached.

The quantitative outcomes (time to acknowledgment of deterioration, time to treatment initiation, escalation of care, and alarm analyses) will be compared with qualitative themes from the staff and patient questionnaires and interviews using a triangulation approach for a better understanding of the effect and implementation of continuous vital sign monitoring. A joint display table will be developed aligning key quantitative results with corresponding qualitative findings, identifying areas of convergence, complementarity, or divergence.

This integrated mixed methods approach aims to facilitate a comprehensive understanding of the usability, acceptability, and practical implications of the Portrait Mobile system.

### Data Governance and Management

The study data will be extracted by a staff member employed as a business intelligence research analyst at Chelsea and Westminster Hospital National Health Service Foundation Trust. These data are stored on secure trust servers, with access restricted to the core study team. These data will be retained for 15 years in accordance with trust policy. An audit trail is available in the EPR.

Each participant will be assigned a unique trial ID during randomization. Variables extracted will include demographics (eg, date of birth, sex, and ethnicity), medical and medication history, vital signs, blood tests, transfer of care, and length of stay. Missing data will not be imputed and will be analyzed as available.

## Results

This manuscript reports the approved study protocol for the WARD-AMS trial, which received favorable ethics approval in April 2024. A total of 200 patients meeting the inclusion and exclusion criteria were successfully randomized, recruited, and completed the period of continuous vital signs monitoring. All patients completed the baseline questionnaire; as of December 2025, a total of 92 patients have participated in the telephone interview. In addition, 50 staff members completed the questionnaire, of whom 27 consented to participate in the interview. As of December 2025, 10 staff members have been interviewed. All data collection is projected to be completed by February 2026; thereafter, it will be cleaned, interviews transcribed, and prepared for data analysis. Results will be finalized with a manuscript submitted for publication by mid-2026.

## Discussion

### Expected Findings

The WARD-AMS study is expected to demonstrate that continuous vital sign monitoring of postoperative patients in a general surgical ward reduces the time from device-defined physiological deterioration to the first objective escalation event (acknowledgment or clinical action) compared to standard-of-care intermittent monitoring. Quantitative analysis will evaluate the changes in response latency, time to treatment, and escalation of care. Qualitative analysis will focus on contextualizing these outcomes by exploring staff and patient experiences. Together, these findings will clarify how continuous vital sign monitoring influences patient outcomes to understand better how to implement these technologies into existing clinical workflows.

### Comparison With Prior Work

Previous observational and before-and-after studies have set a foundation to build on. These study designs and systematic reviews are limited by heterogeneity in device and sensor technologies, alerting protocols, escalation pathways, and staff engagement practices [12,13,34,35]. The COSMOS pilot prospective study evaluated alerting parameters; however, it was not powered for achieving statistical significance [20]. Our study allows for a more comprehensive assessment of the clinical benefit and implementation feasibility in a real-world setting.

### Alarm Fatigue and Implementation

A key area of analysis will involve alarm characteristics, including their frequency and clinical relevance and configuration of alarm thresholds. Understanding this will provide critical insights into addressing alarm fatigue, a known limitation of critical monitoring systems [12,13]. Qualitative data on staff perceptions and experiences will highlight reasons contributing to cognitive burden, such as a high number of false-positive alarms or unclear escalation pathways. These findings will further clarify and provide recommendations on how to integrate these technologies into general wards, improving usability, minimizing disruptions, and maximizing patient outcomes.

### Strengths and Limitations

The strengths of the WARD-AMS study include the randomized design and prospective real-world deployment in an active general surgical ward. The study is being conducted in 2 separate wards, which captures variability in management and staff practices. In addition, both quantitative and qualitative data will be analyzed.

The limitations include the study being conducted in a single center with established escalation pathways. However, these are representative of a typical National Health Service surgical ward, which uses the National Early Warning Score 2 as a track-and-trigger threshold system supported by critical care outreach nurses, and therefore, the results are transferable to similar settings. Lack of blinding of clinicians in the alerting arm may introduce the possibility of performance and detection bias. However, as continuous vital sign monitoring is intended to modify clinical response behavior, staff awareness will reflect real-world practice in assessing implementation and effectiveness.

### Future Directions and Dissemination

Overall, this study expects to lay a robust foundation for larger multicenter evaluation across diverse hospital settings and specialties. Results will be disseminated through peer-reviewed publications, conference presentations, and engagement with clinical innovation teams to support broader adoption and promote safer, more responsive, and equitable health care.

## Supplementary material

10.2196/81558 Multimedia Appendix 1 Supplementary figures, including the study design flowchart and patient and health care staff questionnaires and interview guided questions.

## Abbreviations

bpm: beats per minute
COSMOS: Continuous Vital Sign Monitoring in Surgical Wards
EPR: electronic patient record
HDU: high-dependency unit
ICU: intensive care unit
IV: intravenous
RRT: rapid response team
