# Supplementary material for: Wearable Continuous Vital Sign Monitoring Study (WARD-AMS) to Detect Clinical Deterioration in Postoperative General Surgery Patients: Protocol for a Randomized Controlled Trial
Source: JMIR Res Protoc. 2025 Dec 19;14:e81558. doi: 10.2196/81558 (PMC12716825; doi:10.2196/81558)

**Multimedia Appendix 1**

**Figure S1: Study design flowchart**
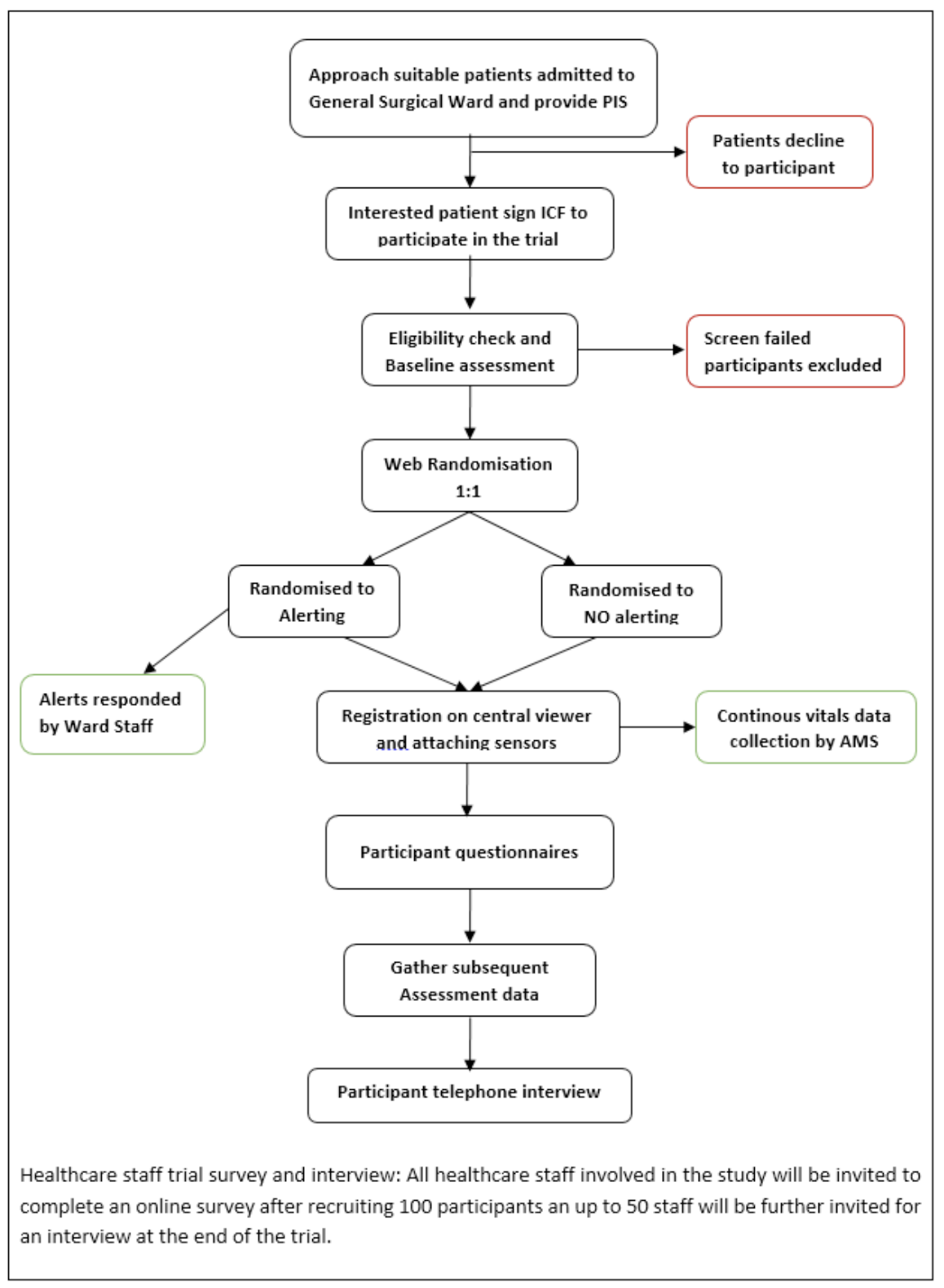


**Figure S2: Healthcare staff questionnaire**
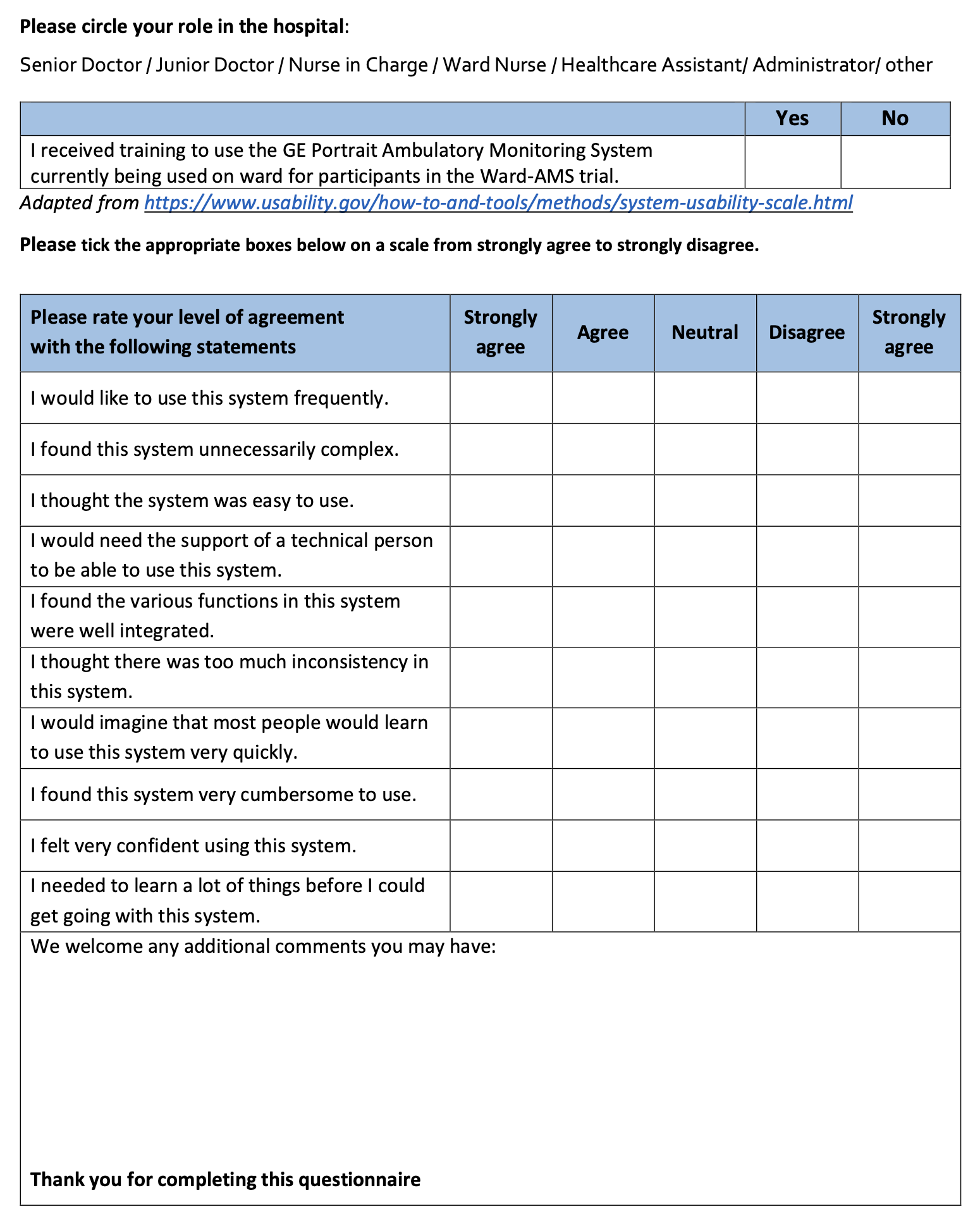


**Figure S3: Healthcare staff interview guided questions**
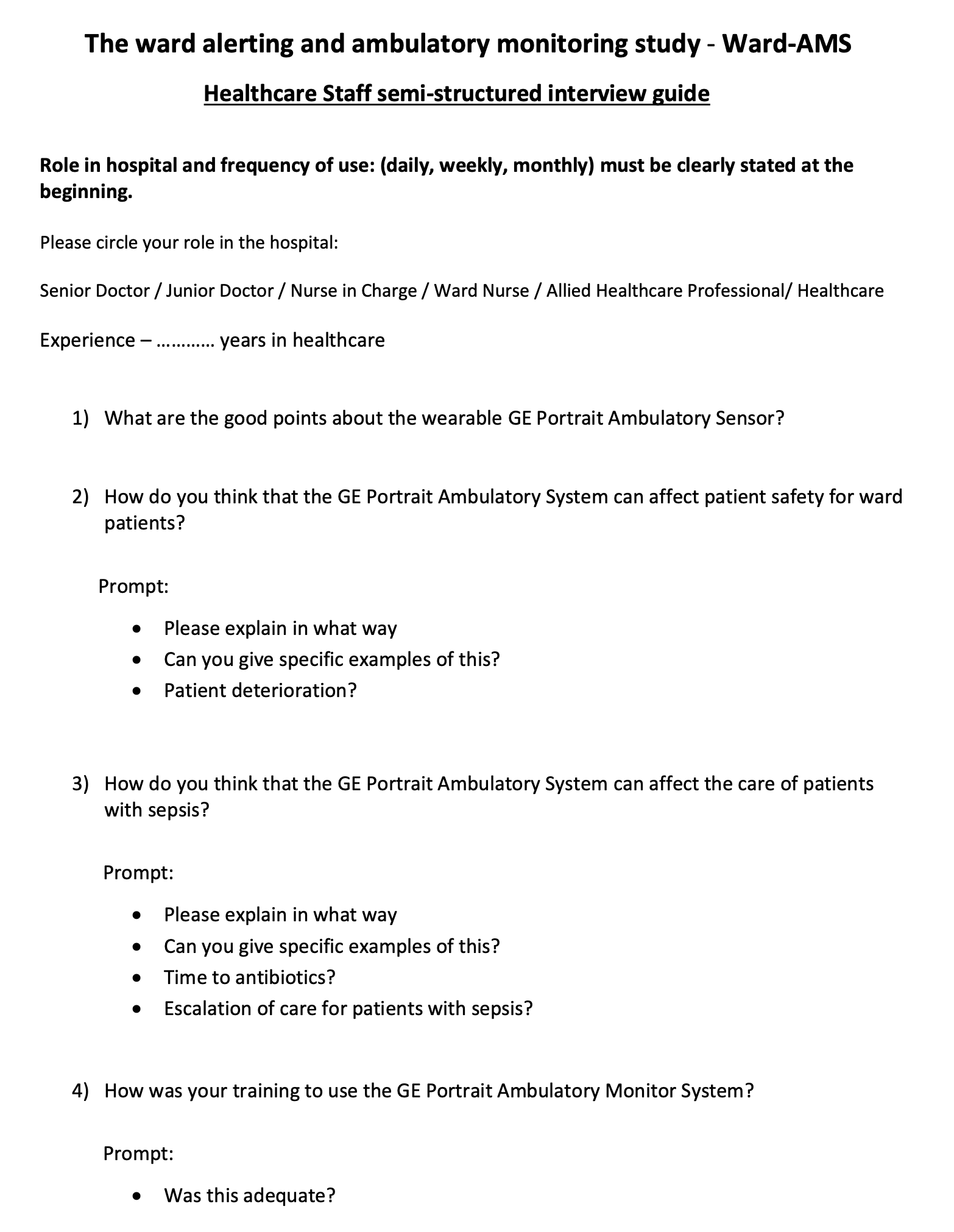


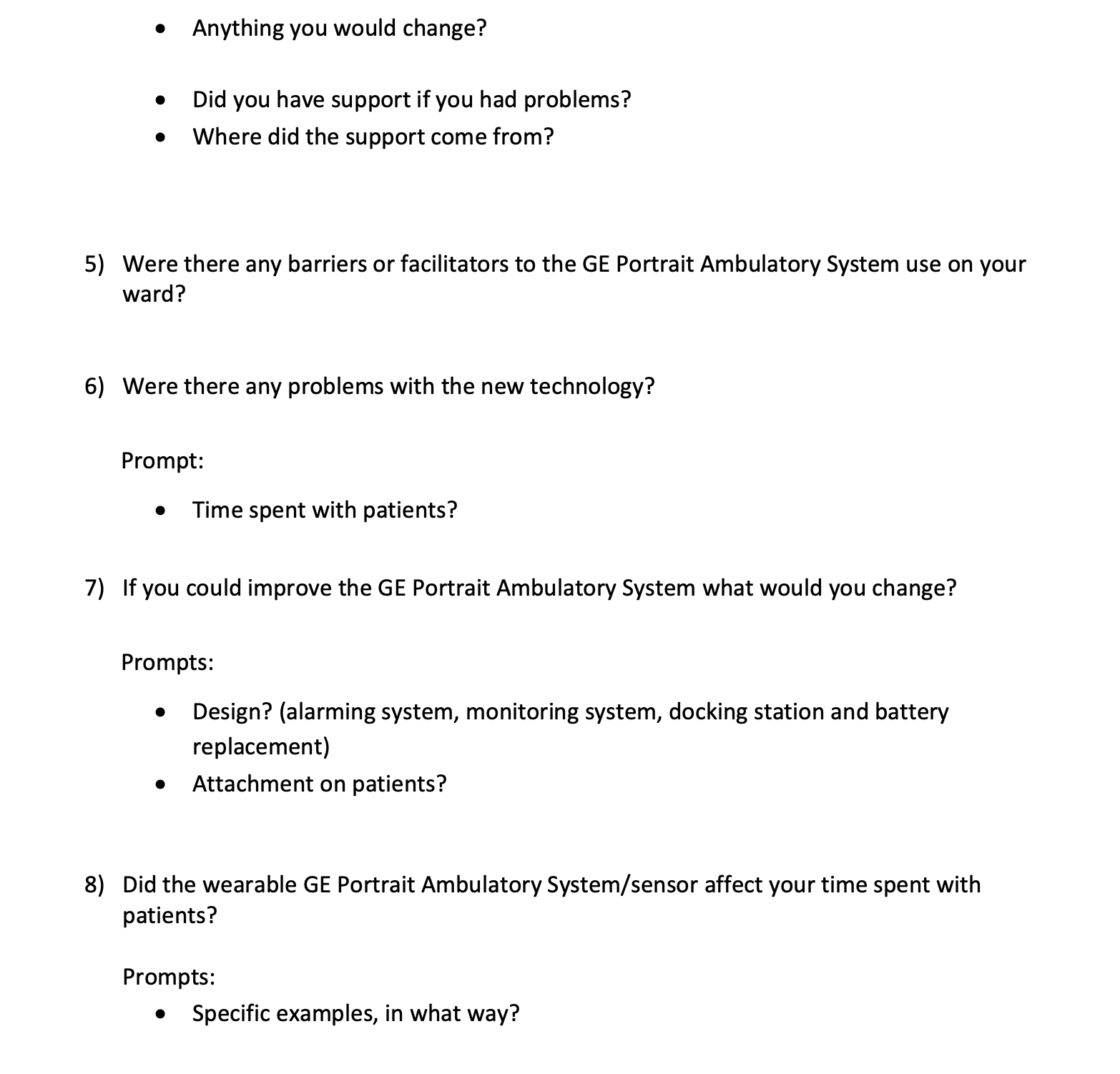


**Figure S4: Patient questionnaire**


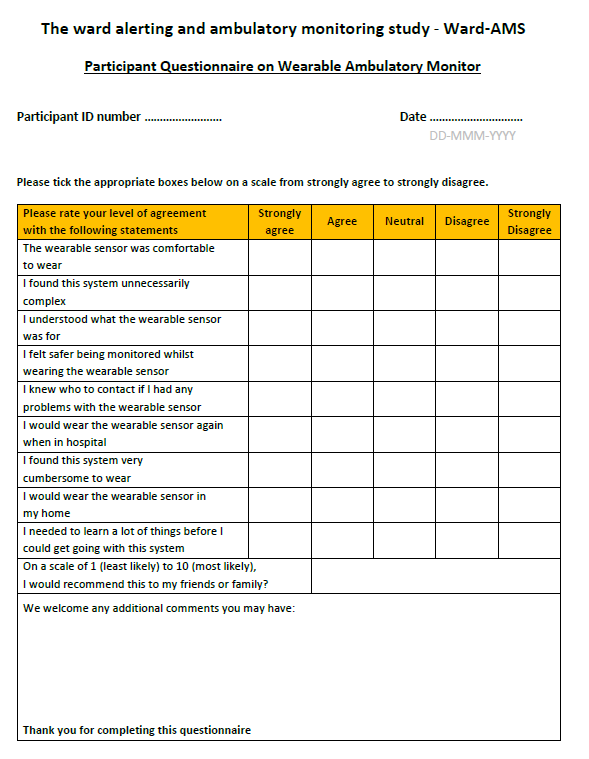


**Figure S5: Patient interview guided questions**
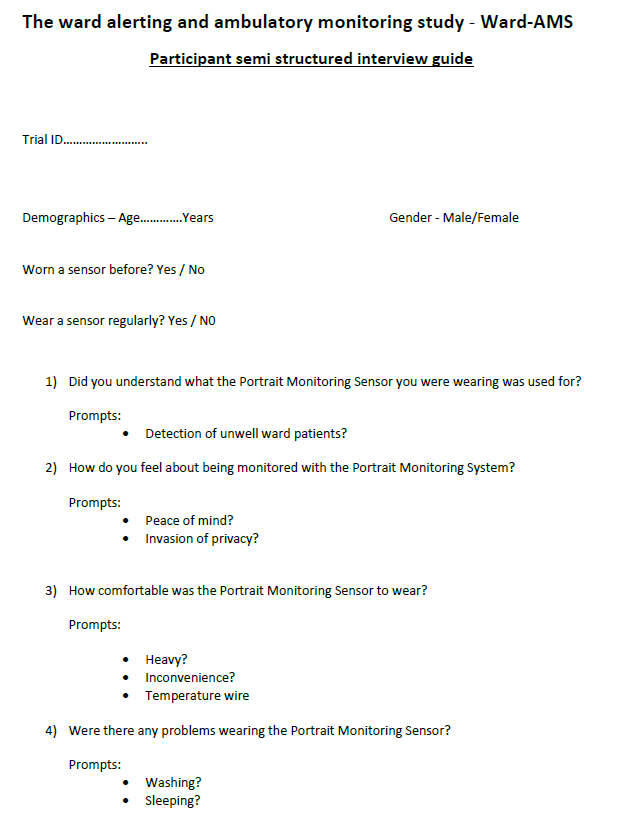


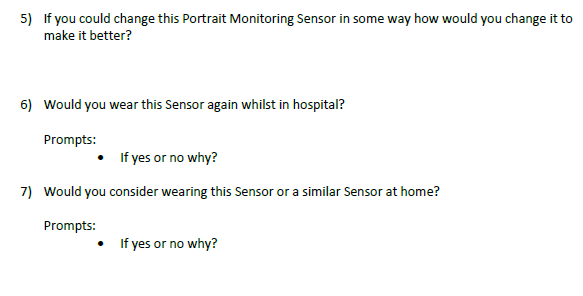

Supplement: Multimedia Appendix 1 [file resprot-v14-e81558-s001.docx]
